# Sialidases as Potential Therapeutic Targets for Treatment of a Number of Human Diseases

**DOI:** 10.3390/ijms26178733

**Published:** 2025-09-08

**Authors:** Cara-Lynne Schengrund

**Affiliations:** Department of Biochemistry and Molecular Biology, The Pennsylvania State University College of Medicine, Hershey, PA 17033, USA; cxs8@psu.edu

**Keywords:** sialidase, neuraminidase, sialic acid, neuraminic acid, gangliosides, sialoglycoproteins

## Abstract

Four human sialidases (hNEUs, E.C 3.2.1.18) have been identified. Each is an exosialidase identified as either NEU1, NEU2, NEU3, or NEU4. They exhibit differences in structure, subcellular distribution, substrate specificity, and the diseases with which they are associated. Similarly, microbial sialidases (NAs) may catalyze the release of sialyl residues from the same sialoglycoconjugates as hNEUs, even though they have low sequence homology with human NEUs. Use of sequence homology, plus the crystalline structure of human NEU2, has provided researchers with the basis for developing inhibitors that may differentiate between them. While microbial-induced diseases that use sialidase to complete their infectious cycle have been the driving force behind interrogation of possible NA inhibitors, errors affecting expression of functional hNEUs and their correlation with clinical problems has led to study of the sialidases per se. Information gained about sialidase structure, function, mechanism of action, mutations affecting expression, and their role(s) in disease, has provided the information about the different sialidases needed for development of specific therapies.

## 1. Introduction

Sialidase participates in the maintenance of a cell’s sialoglycoconjugates, and errors affecting its expression contribute to diseases seen phenotypically such as fibrosis, cancer, and diabetes and in neural development/function. Humans express four distinct human exo-sialidases (neuraminidase, NEU, E.C.3.2.1.18), each capable of catalyzing the release of sialic acid residues (hence use of the term sialidase) from sialylated compounds. Each hNEU has been identified, cloned, and named NEU1 [1], NEU2 [2], NEU3 [3], and NEU4 [4]. Although differences in subcellular location, substrate specificity, pH optimum, and association with physiological problems have been ascertained for each, they are not absolute (see Table 1). Expression levels also differ, with NEU1 expression greater than that of NEU3 and NEU4, while NEU2 expression is lowest in human tissues [5]. The close association of sialidase (NEU3) activity with both its ganglioside substrates and caveolin in plasma membrane microdomains [6] supports the concept that it is able to affect cell function by acting on cell surface sialylated moieties that may be involved in cell signaling [7] (for a review, see [8]). Alterations in NEU3 activity underlie its role in diseases such as fibrosis and cancer (e.g., [9,10]), as well as in neuronal development/function [11,12,13]. For a general review about NEU3, see Miyagi and Yamamoto [14]. While the emphasis, when discussing a specific problem, may focus on a specific hNEU, it should be understood that more than one hNEU may contribute to the resultant changes.

**Figure 1:**
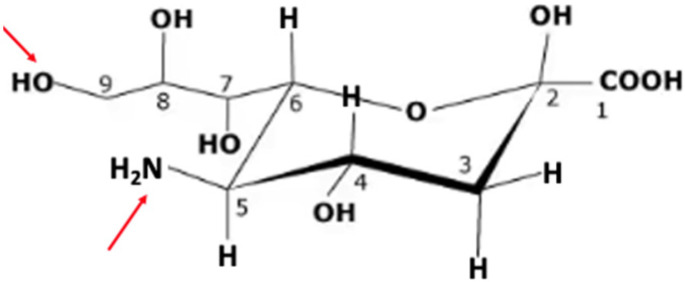
Neuraminic acid (Neu). The IUPAC name for Neu is (4S,5R,6R)-5-amino-2,4-dihydroxy-6-[(1R,2R)-1,2,3-trihydroxypropyl]oxane-2-carboxylic acid. It does not occur naturally, but a number of its derivatives can be found. The arrows in red indicate the site at C5 that is modified by N-acetylneuraminate synthase in humans to yield 5N-acetylneuraminic acid (Neu5NAc) and C9, which can be O-acetylated by the action of sialic acid O-acetyl-transferase identified as capsule structure 1 domain containing 1 (CASD1), catalyzing formation of Neu5NAc9OAc [46].

Sialic acid, the compound released by the action of sialidases, was first characterized by Blix and Gottschalk [38] in mucins isolated from salivary glands, hence the name sialic acid. Some years later it was characterized by Klenk [39], who identified it in preparations from brain, where it is found in highest concentration in the gray matter [40], and who called it neuraminic acid. Subsequently the authors agreed [41] that the unsubstituted compound should be called neuraminic acid (Neu, Figure 1) while substituted versions would be called sialic acid. In human glycoconjugates, the sialic acid most commonly found is 5-N-acetyneuraminic acid (Neu5NAc), while small amounts of Neu5NAc9OAc can also be found [42,43]. The Neu5N-glycolyl found in people is presumably due to that obtained from food as people lack the enzyme needed to catalyze hydroxylation of the CMP-NeuAc needed for its synthesis [44]. The development of methods to synthesize specific acetylated sialic acids (e.g., [45]) for use as standards for the identification of specific sialic acids should help researchers define their biological effects.

Gangliosides, sialylated glycosphingolipids, can be found in lipid rafts [47,48] on the outer surface of cell membranes, with their carbohydrate moiety protruding into the glycocalyx [49], on the outer surface of the plasma membrane [50], and with the lipid portion interacting with the membrane’s lipid and protein components. Changes in either the carbohydrate or lipid composition [51] of the ganglioside can affect its interaction with membrane proteins. Such a change can occur when the carbohydrate portion of the ganglioside interacts with an extracellular component, thereby affecting proteins, including those involved in signal transduction (e.g., [52]). Sialyl residues on gangliosides can, in some instances, block accessibility to receptors. This can be ameliorated by cleaving them with sialidases. The combination of variability in both the carbohydrate and ceramide composition in gangliosides, allows for fine tuning of their behavior in signal transduction [53]. It is in the fine tuning that the ability of hNEUs to catalyze the release of sialic acid moieties from cell surface gangliosides can affect cell behavior [8].

Ganglioside substrates, a major class of sialylated compounds acted upon by hNEUs, include those with terminal α2-3, α2-6, and α2-8 sialic acid residues, as indicated in Table 2. It can be seen that a single sialic acid moiety linked to an internal Gal on gangliosides having additional external sugar residues is not susceptible to hNEU activity [3]. When looking at results obtained in studies of sialidase activity one should note not just type(s) of sialidase(s) expressed and their possible activity overlap, but the animal used, as results obtained may differ from those found in humans. Support for possible animal-related differences was provided by the observation that mice were able to catalyze conversion of both GM1 and GM2 to the corresponding asialo derivatives in the presence of GM2 activator protein [13], a reaction not found in humans lacking either the β-galactosidase or β-N-acetylhexosaminidase A needed for conversion of GM1a and GM2 to GM3 [54].

## 2. Development of Sialidase Inhibitors

**Figure 2:**
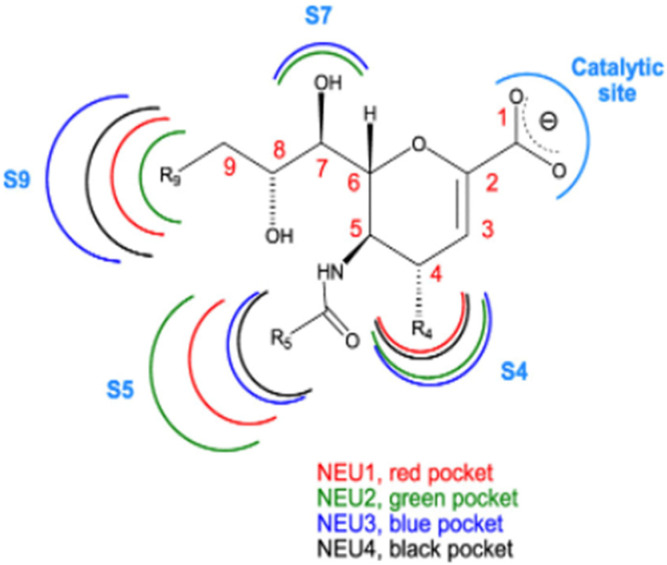
Sites on neuraminic acid modified to give sialic acid-based inhibitors. Copied with permission [Bourguet et al. J. Med. Chem. 2022, 65, 4, 3002–3025] [35]. Larger arcs indicate relative size of effective substituents.

The crystal structure for hNEU2 was used in homology modeling [55] to obtain information about possible binding sites of each hNEU, which in turn was used to develop potential inhibitors of each, based, in part, on how modifications to sialic acid might affect its efficacy as a sialidase ligand. While hNEUs 2, 3, and 4 have 34–40% homology, hNEU1 has only 19–24% [14]. Interestingly, protective protein/cathepsin A (ppCA), found in association with hNEU1, serves both as a chaperone/transporter and to protect hNEU1 from the lysosomal environment [56]. The sites identified for modifying neuraminic acid in order to synthesize hNEU ihibitors are shown in Figure 2 [35] and information about examples of actual inhibitors in Table 3. Comparison of the effectiveness of various sialic acid-based inhibitors at blocking the activity of specific hNEUs allowed researchers to draw conclusions about which site’s modification would block which hNEU activity most effectively. For example, a C9 biphenyl carbamate derivative had high selectivity and potency for NEU3 (µM), while activity of NEU1 and NEU4 was inhibited more efficiently by amide and triazole derivatives, respectively.

The finding that both NEU1 [57] and NEU3 [58] can dimerize supports interrogation of whether blocking dimerization would effectively inhibit their activity [35]. For a general review of sialidase inhibitors see Keil et al. [59].

**Table 3:** Examples of inhibitors for specific mammalian sialidases.

Examples of hNEU Inhibitors	Sialidase Inhibited	Cell Source of NEU	Substrate	Effect
C9-BA-DANA ^1^ [60] ^2^	NEU1	HEK293 cells	4-MU-NANA ^3^	IC_50_ = 10 µM
Neu5Ac2en- OacOMe [61]		Coronavirus ^4^ HCoV-OC43- infected epithelial cells	Viral N protein	Inhibited viral replication.
C5-hexanamido-C9- acetamido-DANA [62]		COS-7 for overexpression of NEU1	4-MU-NANA	K_i_ of 53 ± 5 nM
Oseltamivir [63]		3T3–hEGFR cells	Inhibition of EGF- stimulated sialidase activity	IC_50_ = 4.86 µM
Neu5AcN_3_9N_3_2en [64]	NEU2	*E. coli*-expressed NEU2	Neu5Acα2–6Galβ*p*NP	IC_50_ = 13 ± 3 µM
DANA [65]	NEU3	*E. coli*-expressed NEU3	4-MU-NANA	Ki = 30 µM
5-Acetamido-9-(([1,1′-biphenyl]-4-cabamoyl)-oxy)-DANA [66]		*E. coli*-expressed NEU3	4MU-NANA	IC_50_ = 0.31 µM
2AP [67]		CCl4-induced liver fibrosis in mice	Effect on liver fibrosis	Reduced liver inflammation NEU3
MP [68]		Recombinant NEU3	rhL-TGF-β1	IC_50_ = 0.002 µM
AMPCA [68]		Recombinant NEU3	rhL-TGF-β1	IC_50_ = 0.002 µM
C9-4HMT-DANA [69]	NEU4	*E. coli* expressed NEU4 A 2011	4-MU-NANA	K_i_ = 30 nM

^1^ Abbreviations: C9-BA-DANA, C9-butyl-amide-DANA; DANA: 2-deoxy-2,3-didehydro-*N*-acetylneuraminic acid; C9-4HMT-DANA (5-acetamido-9-[4-hydroxymethyl[1,2,3]triazol-1-yl]-2,3,5,9-tetradeoxy-D-glycero-D-galacto-2-nonulopyranosonic acid); 4-MU-NANA, 2′-(4-methylumbelliferyl)-α-d-*N*-acetylneuraminic acid; 2AP, 2-acetylpyridine; MP, methylpicolinate; AMPCA, 4-amino-1-methyl-2-piperidinecarboxylic acid; rhL-TGF, recombinant human latent transforming growth factor. ^2^ References given cite source of information shown for the NEU inhibitor. ^3^ It should be noted that when 4-MU-NANA is used to measure the K_i_ or IC_50_, the results may differ from those obtained using more natural substrates [70]. ^4^ Desialylation of the coronavirus N protein catalyzed by NEU1 enhances its replication [61].

## 3. Microbial Sialidases

A number of microbes utilize sialidase during the infectious process for such purposes as providing sialic acid residues as catabolites [71], exposing the appropriate cell surface oligosaccharide binding sites [e.g., *V. cholerae* for cholera toxin] and catalyzing their release from those binding sites [e.g., influenza]. The ability to act on cell surface ganglioside and/or sialoglycoprotein binding sites on target cells during the infectious process reflects the fact that the outer surface of the cell’s plasma membrane is coated by a glycocalyx [45] comprised of carbohydrate moieties, including gangliosides and sialoglycoproteins with their carbohydrate portions extending away from the outer surface [72]. The smaller glycosylated portion of gangliosides is found closer to the surface of the cell than the larger sialylated glycoproteins. Perhaps the best-studied sialidase-mediated pathogenic microbial infection is that of influenza virus, in which its sialidase activity participates with hemagglutinin in the infectious process as well as in the release of newly formed virions from sialylated binding sites on the cell surface [73]. It should be noted that sialidase behavior can vary depending on viral subtype [73].

Influenza viruses bind α2-3gal/galNAc and α2-6gal/galNAc-linked sialyl residues [73], which indicates that they could bind those on gangliosides and sialoglycoproteins. The finding that sialidase on the newly synthesized virions had to catalyze the cleavage of the cell surface sialyl moieties on the glycoconjugates to which they are adhered in order to be released and infect new host cells [74] led researchers to develop sialic acid derivatives that might inhibit the viral sialidase more efficiently than it did hNEUs. For a review about influenza sialidase see [75]. Results led to the development of Oseltamivir (Tamiflu) [3R,4R,5S)-4-acetylamino-5-amino-3-(1-ethylpropoxy)-1-cyclohexene-1-carboxylic acid ethyl ester, phosphate (1:1)], discovered by Gilead Sciences in 1995, co-developed with F. Hoffmann-La Roche Ltd., and patented in 1996. Zanamivir (Relenza). The 4-guanidino derivative of DANA, was discovered [76], licensed to Glaxo, and it was Glaxo Wellcome (now Glaxo Smith Kline) who obtained FDA approval for its use in 1999 [77]. Confirmation of the poor ability of the two drugs to inhibit activity of hNEUs was provided by Hata et al. [78], who found that, at 1mM, oseltamivir had little effect on any of the human neuraminidases, while µM quantities of Zanamivir were required to inhibit NEU2 and NEU3. Both drugs were effective on the viral sialidase in low nM concentrations. A third inhibitor of influenza sialidase, Peramivir (Rapivab, (1S,2S,3R,4R)-3-[(1S)-1-(acetylamino)-2-ethylbutyl]-4-(carbamimidoylamino)-2hydroxycyclopentanecarboxylic acid, trihydrate), developed by BioCryst Pharmaceuticals, Inc., gained FDA approval in 2014 [79].

**Figure 3:**
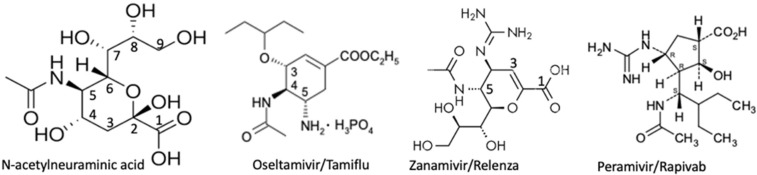
Sialic acid-based inhibitors for influenza sialidase.

Over time, the effectiveness of Oseltamivir and Peramivir has decreased somewhat due to the appearance of a genetic change identified as the H275Y mutation in influenza sialidase [80]. A series of analyses of the susceptability of sialidase associated with cultured samples of the H3N2 variant of influenza to each of the three inhibitors shown in Figure 3, plus a fourth authorized for use in Japan (Laninamivir), was undertaken annually (2010–2020) and again in 2022–2023 in Japan. Results indicate no significant changes in the efficacy of each inhibitor [81]. Despite the reduction in effectiveness seen in some samples from children in the USA, as of September 2024, the CDC was still recommending their use as not all strains of the virus have the mutation and for many people the drugs were still helpful. These drugs may prove useful in the treatment of other ailments, as Zanamivir has been shown to be effective in an animal model of arthritis [82] and Oseltamivir and Peramivir have been seen to reduce mortality in patients with severe COVID-19 [83].

Structures were obtained from the label information for a drug prescribing information insert for Oseltamivir/Tamiflu, GlaxoSmithKline LLC for Zanamivir/Relenza, and accessdata.fda.gov for Peramivir/Rapivab.

Due to the increasing number of influenza subtypes, methods to enhance the effectiveness of Oseltamivir and Zanamivir at inhibiting these subtypes have been, and are being, investigated. One that showed promise was that in which polyglycol was used to link two Oseltamivir molecules together, thereby providing a divalent ligand potentially able to bind more than one active site on the viral sialidase tetramer. The dimers were significantly more effective against three strains of influenza than Oseltamivir alone [84]. The concept of multivalency providing a more effective ligand for a protein that has multiple binding sites for its target ligand is not new [85,86]. The effectiveness of such multivalent carbohydrate ligands as inhibitors for glycoconjugate binding proteins may reflect the fact that, while binding to a single carbohydrate ligand can be weak, binding to the multiple sites that can be found in clusters in the cell’s glycocalyx, can be quite strong [87]. Multivalency has now been used to develop a drug that appears to protect people infected with influenza, as detected by the development of hemagglutinin antibodies, from developing typical flu-associated symptoms. The drug, developed by Cidara Therapeutics and named CD388, is designed to be used prophylactically and to cover a broad spectrum of influenza strains. It is a multivalent derivative in which an average of 4.5 dimers of Zanamivir [88] are linked to lysine residues on the carrier. To prolong the half-life of the multivalent ligand, the surface of the Fc portion of the CH1–Fc hybrid domain of human IgG1, engineered for extended half-life [89], was used as the carrier. The spacing of Zanamivir dimers was such that they could bind to either adjacent sialidase tetramers on the same or nearby virions [88]. Animal tests have indicated that the drug was effective against a number of different strains of viruses, including influenza B. Efficacy was corroborated by results from a small preliminary study in which people were given either CD388 or placebo five days prior to exposure to the virus. A significant reduction in the development of symptomatic infection was seen, with only 17.6%, of those given the drug five days before exposure to virus expressing flu symptoms, while 60% of those given placebo became ill [90]. Cidara plans to start long-term clinical efficacy trials in early 2026 at the start of flu season in the southern hemisphere.

Interestingly, conversion of the NEU2 inhibitor Neu5AcN_3_9N_3_2en to Neu5Gc9N_3_2en produced an inhibitor of *V. cholerae* neuraminidase [64] that effectively prevented its conversion of intestinal gangliosides to GM1 [91], the receptor for cholera toxin [92]. Influenza virus and *V. cholerae* are just two of a number of pathogens that utilize sialidase activity during the infectious process (for some examples see Table 4). With the mounting evidence for the efficacy of multivalent Zanamivir for the prevention of influenza, it is anticipated that researchers will determine whether multivalency enhances the effectiveness of monovalent drugs at inhibiting infection by other microbes.

An alternative approach to the use of chemical inhibitors for respiratory infections is the possible use of an inhaled bacterial sialidase to catalyze removal of the sialic acid residues from molecules on the surface of respiratory epithelial cells, thereby blocking adherance of respiratory viruses to their target cells (e.g., [93]). Conditions for the use of an inhaled bacterial sialidase against influenza and parainfluenza viruses have been studied [94] and its use against additional pulmonary problems that can make a person more susceptible to viral infection, such as chronic obstructive pulmonary disease, is also being investigated [95]. This approach could eliminate the possibility that chemical inhibitors developed to treat a microbial infection would inhibit an hNEU, as observed when antibodies prepared against sialidases produced by either *C. perfringens* or influenza virus were found to inhibit hNEU3 [96].

**Table 4:** Examples of pathogens using sialidase during infection and, when known, potential inhibitors.

Pathogen	Cells Infected	Disease In people	Binding Site(s)	Inhibitor	Inhibitor Efficacy
NDV ^1^ HN ^2^ [97]	Lung epithelia.	Mild flu-like in humans, deadly in birds.	α2-3- and α2-6-linked sialic acid [98].	4-trifluoro-acetamido-N-trifluoroacetyl-DANA,	Viral yield reduction assay IC_50_ = 0.03 µM [99].
MuV HN [100]	Multiple types.	Mumps complications including e.g., deafness, meningitis, and infertility.	Trisaccharide receptors: α2-3SL, α2-3SLN, α2-3sLe^x^, and oligo-GM3 [101].	Milk-derived sialoglycopeptides with short glycans having α2-3-linked terminal sialic acids.	Inhibited infection by different strains; based on sialic acid concentration IC_50_ = 0.2–1 mM [100].
Influenza A and B [102]	Pulmonary epithelia.	Flu.	Human strain A, α2-6-, strain B α2-3- and α2-6-linked sialic acid [103].	Oseltamivir. Zanamivir. Peramivir.	Substrate 4-MU-NANA, IC_50_ = 0.78 nM, IC_50_ = 2.08 nM, IC_50_ = 0.66 nM, and influenza isolate 74 A H3N2 [81].
Dengue serotype 2 [104]	Blood, skin, and liver.	Fever, and possibly headache, muscle or joint pain, nausea, vomiting, pain behind the eyes, swollen glands, rash.	Viral NS1 affects transcription of NEU1-4, suggesting that they help with viral Uncoating, replication and intracellular trafficking [105].	Not a viral sialidase.	Dengue affected expression of all hNEUs [105].
*Vibrio cholerae* [106]	Intestinal mucosal cells.	Cholera.	Di-and trisialo-gangliosides terminal sialic acid-linked α2-3 and α2-8 [107].	Neu5Gc9N_3_2en.	Neu5Acα2-3Galßρ NP, IC_50_ = ~18 µM Neu5Acα2-6Galßρ NP, IC_50_ = ~13 µM [64].
*Gardnerella vaginalis *[108]	Genital tract.	Vaginal irritation and increases probability of pre-term birth.	Mucosal sialoglycans [109].	10 mM Zanamivir.	Cell invasion decreased ~50% [110].
*C. perfringens *type F ^3^, Nans I, J and H. I provides most of the exosialidase activity [111].	Intestinal enterocyte type cells	Food poisoning and chronic non foodborne gastrointestinal diseases.	Linkage cleaved: I, α 2-3; J, α 2-6; and H, α 2-8 [112].	7-(3,4-dihydroxyphenyl)-5-hydroxy-1-(3- hydroxy-4-methoxy phenyl) hepta-1,4,6- trien-3-one (individual Nan not identified).	Substrate 4MU-NANA IC_50_ = 0.5 ± 0.07 µM for Nan I [113].
*S. pneumoniae* Nans A, B, and C [114]	Lung and epithelial cells.	Pneumonia, meningitis, and sepsis.	Surface protein A functions as an adhesive while sialidase degrades mucus, providing sialic acid as a bacterial nutrient [115].	Zanamivir for NanA. 1 mM Oseltamivir for NanA.	K_i_ = 0.72mmM using MU-NANA [116]. Inhibited in vivo *S. pneumoniae* viability [115].
*C. sordellii*, NanS [117]	Soft tissue and intrauterine infections.	Toxic shock and sepsis; low survival [118].	Acted on cervical cell sialoglycoconjugates enhancing suscept-ability to bacterial toxins [117].		
*Porphyromonas gingivalis* [119]	Mouth.	Severe periodontitis.	Submaxillary glycoproteins.	Zanamivir.	Inhibited attachment and invasion [119].

^1^ Abbreviations: NDV, Newcastle disease virus; MuV, mumps virus; Nan followed by a capitol letter identifies the type of microbial sialidase; bacteria names are italicized; C., Clostridial; and S., Streptococcus. ^2^ HN indicates a single glycoprotein with both hemagglutinin and sialidase activity. Research indicates that H mediates binding and N cleaves sialic acid from progeny virus, promoting release of newly formed virions during the budding process [120]. The reason for looking at the inhibitors of infection by the mumps virus is the ineffectiveness of the vaccine against some of the strains. Hemagglutinin has 3 sialic acid binding sites [121] so multivalent inhibitors might be used to block its function in binding. ^3^ *C. perfringens* type F was previously known as *C. perfringens* type A [122].

## 4. Examples of Diseases Involving hNEUs

Characterization of the structure of the four different human sialidases and an understanding of their functions, has led to investigation of how alterations in their specific activity affect disease. Studies of each have shown they function in a variety of diseases, ranging from those caused by exposure to environmental agents such as coaldust or asbestos (e.g., [123]), to mutations that affect expression/activity of the NEU. Examples of the diseases discussed, and for which hNEUs can be a factor, include pulmonary fibrosis [9,23], atherosclerosis [124], diabetes [125], specific cancers [30], and neuronal development/function [126]. Examples of each and of possible therapeutic sites based on a NEU or NEUs follow.

### 4.1. Fibrosis

Coaldust and asbestos-induced pulmonary fibrosis are examples of environmentally induced diseases in which both NEU1 and NEU3 have been shown to have a role [9,23]. NEU1 presumably acts by catalyzing release of sialic acid from the membrane-tethered mucin, MUC1, thereby enhancing cell adhesion [127] while activated NEU3 may act on ganglioside substrates which in turn can affect tumor growth factor-ß1 (TGF-β1 [128]). This is of interest because TGF-β1 has been reported to be a major driver of lung fibrosis via the promotion of the differentiation of fibroblasts into myofibroblasts that produce excessive extracellular matrix that can contribute to deteriorating lung function [129]. The finding that TGF-ß1 can initiate a series of down-stream events forming a phospho-Smad3/MUC1-CT (MUC1-cytoplasmc tail) and MUC1-CT/β-catenin nuclear complex capable of enhancing conversion of alveolar epithelial type II cells and fibroblasts to myofibroblasts provides an explanation for its function in pulmonary fibrosis [130]. Increased MUC1 is found in both bronchoalveolar lavage and serum from those with pulmonary fibrosis, while it is present in less than 10% of serum samples from patients with chronic lung diseases [128]. After oropharyngeal instillation of silica into the lungs of hMUC1 transgenic and MUC1 KO mice, elevated levels of the covalently linked extracellular glycosylated α-subunit of MUC1 were found in the lungs of the transgenic animals and their lungs were protected from fibrosis [128]. Release of the extracellular domain of MUC1 allows it to act as a decoy barrier to external pathogens, while the bioactivated CT portion can act as an anti-inflammatory molecule in a number of airway infections [131]. Combining these observations indicates how the activities of both NEU1 and 3 could affect the clinical symptoms seen in environmentally induced pulmonary fibroses [132].

Some patients with idiopathic pulmonary fibrosis (IPF) have abnormally high levels of NEU3, which can upregulate active serum TGF-β1. TGF-β1, in turn, can upregulate NEU3 expression by decreasing its degradation and up-regulating its translation [133]. Translation is upregulated by TGF-β1 enhancing the binding of DEAD box helicase 3 (DDX3) to a common 20-nucleotide motif present on a total of 180 mRNAs, including that for NEU3. Reduced DDX3 reduced TGF-β1-enhanced NEU3 synthesis. Mice lacking NEU3 had very little fibrosis when treated with bleomycin to induce it [134].

Using a mouse model of kidney fibrosis, induced using unilateral ureteral obstruction (UUO), Xiao et al. found that NEU4 activity was upregulated, a finding also seen in patients with renal fibrosis [134]. Results from the studies of kidneys from male NEU4^−/−^ mice, UUO-induced to develop kidney fibrosis, showed that the epithelial to mesenchymal cell transition was attenuated, as was the production of pro-fibrotic cytokines. NEU1 appears to have a similar effect on kidney fibrosis [135].

Patients with non-alcoholic fatty liver disease (NAFLD) may also develop fibrosis [136]. The finding that NEU3 was elevated in patients with idiopathic pulmonary fibrosis led to study of its potential role in NAFLD. Using mice treated with CCl_4_ to induce liver inflammation and fibrosis, treatment with the NEU3 inhibitor 2-acetylpyridine was found to ameliorate the symptoms [67]. Interestingly, the intestines of patients with inflammatory bowel disease, many of whom also develop fibrosis [137], were found to contain 8-fold more NEU3 than controls, while the concentration of GD1a, an NEU3 substrate, was 1/3 [138]. All of these results support the interrogation of whether specific hNEUs, or components in the pathways they affect, might be attractive targets for the development of therapies to treat specific types of fibrosis.

### 4.2. Atherosclerosis

Atherosclerosis, accumulation of plaque within arteries, contributed to by hypoxia-induced autophagy and macrophage inflammation [139], is affected by hNEU1, with the level of NEU1 gene transcripts in lipofibrous plaques significantly increased compared with that in healthy tissue samples [140]. The fact that NEU1 can affect insulin resistance [141], lipid metabolism [142] and inflammatory responses [24], has made NEU1 a likely candidate for study. Results from the analysis of the development of atherosclerosis in *Neu1^hypo^Apoe^−/−^* mice indicate a decrease in aortic sinus atherosclerosis when compared with *Apoe^−/−^* mice [143]. Additional studies of the possible effect of hypoxia on hNEU1 have indicated that hypoxic conditions enhanced the desialylation of the autophagy protein ATG_5_ and promoted formation of an ATG_5_–ATG_12_–ATG_16L_ complex, enhancing autophagosome formation. Hypoxic conditions were accompanied by translocation of hNEU1 from lysosomes into the cytoplasm and an up-regulation in its activity towards sialylated ATG_5_. Inhibition or knock-down of hNEU1 inhibited desialylation of ATG_5_ and inhibited the hypoxia-induced autophagy and cellular inflammation seen in atherosclerosis [144].

mRNAs for hNEU3 and hNEU4 were found to be 20-fold higher in lipofibrous plaques than normal tissue (human thoracic samples) and has been suggested to be a specific marker of atherosclerotic plaque instability [140]. NEUs1 and 3 were shown to trigger atherosclerosis by desialylating ApoB100 in low-density lipoproteins (LDLs), thereby increasing their uptake via the asialoglycoprotein receptor 1 present on human macrophages as well as aortic root lesions in mice [124]. Genetic inactivation or pharmacological inhibition of NEUs1 and 3, but not NEU4, significantly delayed the formation of fatty streaks in the aortic root, without affecting plasma cholesterol and LDL levels in *ApoE^−/−^* mouse models of atherosclerosis. Combined, these results support the hypothesis that the action of sialidase on ApoB100 might be the reason that some of the ~50% of people develop atherosclerosis despite having LDL levels thought to be in the normal range [145]. To interrogate this possibility one could (1) test individuals who have an atherosclerotic event, despite having a history of normal LDL levels, for the presence of desialylated ApoB100; (2) develop a simple method to quantify the amount of desialylated circulating ApoB100 in order to identify individuals for whom uptake of LDLs by macrophage could lead to atherosclerosis; and (3) develop an effective protocol for inhibiting desialylation of ApoB100 to retard LDL accumulation by macrophage.

### 4.3. Diabetes

As early as 2003 Sasaki et al. [146] reported that overexpression of the “plasma membrane associated sialidase” (NEU3) attenuated insulin signaling. Support for this was provided by the observation that transgenic mice overexpressing the human ortholog of NEU3 developed diabetic characteristics, such as hyperinsulinemia, after 18–22 weeks [146]. Analyses indicated that phosphorylation of the insulin receptor (IR) was reduced in response to insulin, while NEU3 tyrosine phosphorylation increased, as did its association with the growth factor receptor-bound protein 2, which activated the NEU3, thus enhancing the negative regulation of insulin signaling. Interestingly, the accumulation of GM1 and GM2, possible products of the transgenic NEU3, inhibited IR phosphorylaton in vitro [146]. Subsequent studies have found that KO of the *NEU2* gene in mice altered sialylated glycoproteins needed for lipid metabolism, impaired muscle function, and led to diabetes [26]. NEU1 appears to reverse insulin resistance in type 2 diabetes [141]. Experimental results indicate that insulin receptor ß (IRß) is activated by a G-protein-coupled receptor (GPCR)-signaling platform potentiating NEU1 and matrix metalloproteinase-9 (MMP-9) cross talk on the cell surface. The activated NEU1 catalyzes release of α2-3 linked sialyl resides from IRß, allowing association of IRα and IRß subunits and activation of tyrosine kinase [147]. For an explanation of the structure and function of the insulin receptor see [148].

### 4.4. Cancer

Evidence for a correlation between sialidase activity and cancer was reported more than 50 years ago [149,150,151,152]. Since then, the four hNEUs have been identified and research is now focusing on their individual contributions to the oncogenic process. As an example, over time, evidence has been found indicating that NEU3 promotes cell invasion by renal cell carcinoma [153], head and neck squamous cell carcinoma [154], and glioblastoma cells [155]. As hNEUs have been studied in more cancers, their possible functions are beginning to be understood, such as those of NEUs1 and 3 in bladder cancer tissue cells [30]. The finding that knock down of NEU3 results in decreased invasiveness, reduced phosphorylation of ERK and P13K and decreased expression of the androgen receptor provides insight into how elevated NEU3 activity may contribute to oncogenicity in the bladder cancer tissues studied [30]. The fact that tissues from different bladder cancers may not express the same growth factors and receptors [30] supports the need for a general data bank in order to optimize the use of personalized medicine in their treatment. Support for this suggestion is provided by the fact that much of the information used by some of the researchers, whose data are cited in Table 5, was drawn from a variety of data bases, such as ONCOMINE [156], the Cancer Genome Atlas (TCGA, accessed through the National Cancer Institute’s Genomic Data Commons (GDC), the Gene Expression Omnibus (GEO, accessed via the NCBI GEO homepage), the Cancer Cell Line Encyclopedia (CCLE, data can be downloaded from the DepMap portal), and the Human Protein Atlas (HPA).

## 5. Sialidase in Neuronal Development/Function

### 5.1. Development

Analysis of brains from chickens has indicated that sialidase activity is expressed in 13-day old embryonic chicken brains and that its activity towards endogenous substrate increases through 3 months post hatching, while that towards added ganglioside substrate decreases [169]. In people, ganglioside sialidase has been detected between fetal weeks 15 and 20, reaching about half that found in adults at term and increasing thereafter to that found at about five years of age [170]. Neuronal development occurs over time and research has shown that each hNEU contributes to changes that occur. In terms of development, several cell adhesion molecules, including E-cadherin, integrins, and catenins, are sialylated glycoproteins [171] and aberrant sialylation can disrupt interactions with their receptors, such as those needed for stem cell differentiation [36]. Murine NEU4 catalyzes the degradation of the polysialic acid which associates with neural cell adhesion molecule (polySia-NCAM) affecting not just neurite growth but synaptic plasticity, and cell migration as well [172]. Results from studies of fish (Tilapia) indicate that, when they are grown in the absence of sunlight, expression of *NEU4* mRNA as well as retinal differentiation markers was upregulated [173]. Addition of Tilapia NEU4 to two different neuroblastoma cell lines decreased sialic acid levels in nuclear glycoproteins and glycolipids, accelerated neurite formation, and enhanced acetylcholinesterase activity, supporting the hypothesis that NEU4 accelerates differentiation. NEU4 activity in Japanese rice fish was relatively low in the brains of embryos and then rose rapidly 3–13 days after birth [174]. In contrast, NEU3 expression was high in embryos and decreased after birth. The decrease in NEU3 expression after birth appears to coincide with neuronal differentiation and the appearance of the more complex gangliosides GD1a, GD1b. and GT1b [175,176,177], known NEU3 substrates [178]. Those gangliosides, plus GM1a, account for ~75% of the sialic acid found in the adult brain [5,179]. The recent development of a method to isolate both neural stem progenitor cells and oligodendrocyte progenitor cells from the brains of live rats [180] means that developmental changes can be studied in each, as the cells mature in tissue culture.

The observation that induced differentiation of pheochromocytoma (PC12) cells was accompanied by an induction of transcription of NEU2 and that the activity of NEU2 disappeared in differentiated PC12 cells indicates a defined time period for the need for NEU2 during their differentiation [181]. Identification of the interaction of NEU2 with α- and ß-actin [182], coupled with the requirement of sialylated glycoproteins for actin to function [183,184] support the hypothesis that NEU2 may act to remodel actin via desialylation of one or more of the sialoglycoproteins needed for its involvement in neuronal differentiation.

NEU1 acts to stop the migration of hippocampal granule cells by catalyzing release of sialyl residues from cell surface polysialic acid when they reach the innermost surface of the granule cell layer [185]. NEU1 influences microglia by modulating the sialylation of full-length Trem2 (Trem2-FL), a multifunctional receptor that regulates microglial survival, phagocytosis, and cytokine production [186]. When NEU1 is deficient/down regulated, Trem2-FL remains sialylated, accumulates intracellularly, and is excessively cleaved into a C-terminal fragment and an extracellular soluble domain, enhancing signaling capacity. Because NEU1 and Trem2 are implicated in neurodegenerative/neuro-inflammatory diseases, including Alzheimer’s disease and sialidosis, modulating NEU1 activity may present a therapeutic approach to broadly regulate microglia-mediated neuroinflammation. Combined, these findings support the hypothesis that expression of each of the hNEUs correlates with changes in the degree of sialylation of differentiation-related sialoglycoconjugates that function during neural development.

### 5.2. Relation of Sialidase to Neural Transmission

During the neural transmission that is induced by the exposure of rat hippocampal slices to a high-frequency electrical stimulation to induce long-term potentiation, NEU4 activity was found to change within seconds [187] by using a benzothiozolylphenol-based sialic acid derivative to monitor free sialic acid levels [188]. NEU4 can catalyze the cleavage of sialyl residues on the polysialylated neural cell adhesion molecule (polySia-NCAM) which carries a linear homopolymer comprised of 8–90 sialic acid residues linked α2-8 [172]. PolySia-NCAM functions in brain development, synaptic plasticity and learning (for a review see [189]). Genetic or enzymatic manipulations blocking synthesis of polySia-NCAM were found to result in impaired synaptic plasticity and learning that could be ameliorated by the addition of polysialic acid [190].

**Figure 4:**
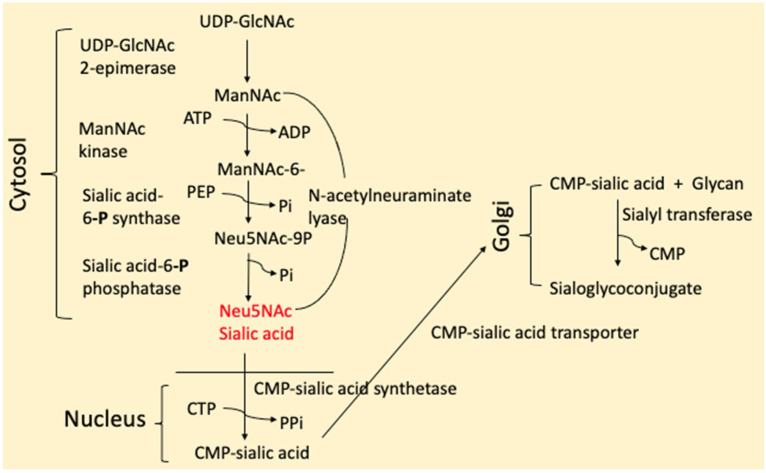
Synthesis of sialic acid and its subsequent incorporation into sialoglycoconjugates.

While not discussed in this review, one must consider not just the expression of hNEUs but the availability of sialic acid, sialyl- and glycosyltransferases and the transport proteins used to synthesize the sialoglycolipids and sialoglycoproteins that NEUs modify as needed (see Figure 4). Some examples are as follows: knockdown of the *ST6Gal1* gene or the use of sialyltransferase inhibitors negatively impacts the efficiency of somatic cell reprogramming [191], failure to synthesize the GM3 induced by a homozygous loss-of-function mutation in the gene encoding GM3 synthase (*ST3Gal5*) results in infantile-onset symptomatic epilepsy syndrome [192], while mutations in the human *ST8SIA1* gene are associated with an increased risk of developing multiple sclerosis [193].

### 5.3. Examples of Sialidase Misfunction in Neural Diseases

Sialidosis: Misfunction of hNEU1 has been implicated in sialidosis, Parkinson’s and Alzheimer’s diseases (PD and AD). Lysosomal NEU1 functions to catalyze the release of sialic acid from sialylated glycoproteins and glycolipids and when it fails to do so, the resultant lysosomal storage of those products is evidenced by clinical problems reflective of the degree of substrate accumulation [194]. Sialidosis, a rare, autosomal recessive disease is clinically divided into two types: in type 1, symptoms of progressive neurological and ophthalmologic problems present in late childhood or early adulthood, while in type 2 symptoms are more severe and present earlier [195]. Interestingly, ethnicity was found to correlate with the expressed phenotype, with Asian patients with sialidosis type 1 tending to have less severe symptoms than Caucasians [196]. This, coupled with the finding of a variety of genetic mutations [197], underscores the need for an understanding of the relationship between genotype and phenotype in order to optimize patient care [198]. Treatment of this error requires increasing the amount of functional NEU1 in the lysosome. The need of NEU1 for protective protein/cathepsin A to assist its entry into the lysosome and, once there, to protect it from degradation presents an obstacle to the use of NEU1 alone [199]. For a discussion of possible treatment approaches see [198].

Parkinson’s disease (PD): In contrast to the rare occurrence of sialidosis, PD is the second most common neurodegenerative disease in individuals ≥50 yrs of age, with an estimated 8.5 million globally affected in 2019 (World Health Org. Parkinson’s disease data: https://www.who.int/news-room/fact-sheets/detail/parkinson-disease#:~:text=PD%20is%20a%20clinical%20diagnosis,of%20over%20100%25%20since%202000, accessed on 9 July 2025)—a number that is expected to reach 12.7–17 million by 2040 [200]. A number of studies have indicated that treatment of animals expressing PD symptoms with isolated GM1 showed an increase in striatal dopamine and enhanced dopamine synthetic capability in residual dopaminergic neurons (e.g., [201,202]) and, when used in a monkey model of PD, improvement in cognitive and motor deficits was observed [203]. Lipid analyses have indicated alterations in expression of gangliosides and that the deficiency in GM1 correlated with Parkinson’s [204]. Subsequent studies of the efficacy of GM1 for the treatment of patients with PD have indicated that it is effective but that its use is problematic [205]. A major problem is obtaining enough GM1 that is guaranteed to be prion free, with sialic acid that is solely NeuNAc, and which is available in large quantities. Use of an in vivo method for the synthesis of GM1 [206] may address the problem of large-scale synthesis. The problem of GM1 transport across the blood–brain barrier [207] might be circumvented by infusing it intranasally, a method found to reduce neurotoxic proteins and restore functional neurons when used in a PD mouse model [126]. To circumvent both problems, *V. cholerae* sialidase was administered intraventricularly to MPTP-treated mice. Its use was based on the premise that the exogenously added sialidase would increase the conversion of di- and trisialogangliosides to GM1. Results indicate that dopaminergic neurons of the substantia nigra pars compacta were also spared by exposure to sialidase, occurred when animals were treated with GM1 [208]. More recently the oligosaccharide portion of GM1 (oligo-GM1) was shown to cross the blood–brain barrier (BBB) and promote recovery in a sporadic PD model in the *B4galnt1^+/^^−^* mouse [209]. Partial loss of expression of B4GALNT1 results in failure to synthesize enough GM2 and the more complex gangliosides, including GM1, causing both motor and non-motor symptoms of PD [210]. Using a cellular model to understand how the oligosaccharide portion might cross the BBB to promote recovery in the *B4galnt1^+/^^−^* mouse, researchers concluded that it was via a paracellular mechanism and that this permitted transport of 20-fold more oligo-GM1 than of intact GM1 [207]. Analysis of the enzymes involved in the metabolism of gangliosides and sialoglycoproteins indicated that NEU4 in lysosomes is decreased in PD and therefore cannot catalyze conversion of more complex gangliosides to GM1. In addition to an increase in GD1a and decrease in GM1, NEU4*^−^*^/*−*^ mice had enhanced astro- and microgliosis as well as motor impairment [211].

Accumulating evidence indicates that chronic inflammation contributes to neurodegenerative diseases and that microglia are involved in the process (for a review see [212]). In response to inflammation induced by exposing microglia to lipopolysaccharide, microglia were found to secrete NEU3 associated with extracellular vesicles that are able to fuse with neurons. There, NEU3 can act to catalyze the release of sialic acid from cell surface sialoglycoconjugates, modifying the neuronal glycocalyx and neuronal connectivity [213]. These results support the hypothesis that, over time, remodeling of the glycocalyx induced by inflammation and the attendant effects on network-level activity of neurons could contribute to neuroinflammatory diseases such as Parkinson’s and Alzheimer’s [213]. If this is proven correct, identification of effective anti-inflammatory agents might provide potential drug targets.

Alzheimer’s: The fact that an estimated 7 million Americans age > 65 yrs are currently living with Alzheimer’s dementia and that number is growing (Alzheimer Disease Assoc, 2024: https://www.alz.org/alzheimers-dementia/facts-figures#:~:text=Over%207%20million%20Americans%20have,older%20(11%25)%20has%20Alzheimer’s, accessed on 9 July 2025), makes this the sixth leading cause of death in 2022 for Americans in this age group. It also underscores the need for an understanding of the disease’s development in order to identify targets for drug development.

NEU1 deficiency in mice induces a spontaneous phenotype of AD-like amyloidosis, while overexpression of NEU1 obtained by injecting NEU1 into the brain of an AD mouse model was conducive to a reduction in amyloid plaques [214]. Early work has indicated that ß-amyloid bound cell surface glycolipids or glycoproteins [215] and that that binding required sialic acid [216]. More specifically, evidence indicates that the association of ß-amyloid with GM1 appears to enhance formation of amyloid plaques [217]. These findings led to interrogating the possibility that added NEU1 might catalyze cleavage of non-ganglioside associated clustered sialyl residues present on the cell surface or cytosol that might interfere with binding to GM1. Support for this hypothesis was provided by the observation that ß-amyloid-induced toxicity could be reduced by binding the ß-amyloid to sialic acid-conjugated dendrimers [218]. Exosomes can carry proteins, including glycosidases, from one cell to another [219]. The proteins can act on compounds in the extra cellular space altering, in the case of sialidases, sialic acid distribution on the surface of the glycocalyx and possibly affecting cell adhesion and invasion (modified from Sanderson et al. [220]). It has been postulated that, over time, this remodeling can gradually uncover amyloid binding sites that affect the progression of AD and which eventually cause neurons to die.

The NEU4-catalyzed removal of sialyl residues from hippocampal polySia-NCAM inhibits neurite outgrowth [172] while NEU3 enhances it [174]. As stated in the discussion of Parkinson’s, activity of extravesicular NEU3 on glycocalyx sialoglycoconjugates can modify network connectivity, affecting network-level neuronal activity [213] and contributing to the problems seen in AD.

While amyloid and tau have been hypothesized as the primary components causing Alzheimer’s [221], other factors are being studied. Based on observations that the ratio of toxic to nontoxic microRNAs predicted sensitivity of ovarian cancer cells to platinum [222], this approach was used to determine whether the ratio of nontoxic to toxic sRNAs might correlate with the neurotoxicity seen in AD [223]. Results indicate that a shift to more toxic sRNAs correlates with neuronal cell susceptibility due to the death induced by survivor gene elimination [223]. The reported observations support the hypotheses that (1) high expression of nontoxic miRNAs protects from neurodegeneration and that increasing the biogenesis of nontoxic miRNAs or blocking that of toxic RNA-induced silencing complex sRNAs might be a viable treatment option for many neurodegenerative diseases, including AD, and (2) that identification of the eliminated survivor genes will further our understanding of symptom development in AD and suggest possible therapeutic targets.

## 6. Conclusions

Research designed to learn about both human and microbial sialidases, their structure, substrate specificity, mechanism of action, behavior in disease, etc. has grown consistently since 1957, the year for which I found the first listing in PubMed using the search word sialidase. This growth in publication has been reasonably steady, with much information now available about sialidase structure, function, cellular location and mechanism of action; however, much is still unknown.

When discussing the various diseases affected by hNEUs, it is evident that more than one isozyme may contribute to the symptoms seen, reflecting limitations in both their substrate specificity as well as their subcellular location. To consider interrogating the best possible treatment for an effect that their expression or lack thereof induces, we need to know more about the primary substrate each NEU acts on, resultant up- and down-stream effects, and how they may alter cell behavior. When looking at cancer, evidence indicates a given hNEU isozyme does not necessarily have the same effect in different tumors. All of these are reasons for establishing a repository for information regarding sialidase, possibly, in part, by combining on-line ones such as those mentioned earlier in this review. Such a repository should be readily accessible and be contributed to by researchers world-wide. In addition to specific facts, it should include connections between observations made. For example, what are the differences that enable an hNEU to induce one response in one type of cancer and a very different response in another, or which enable different results to be seen in animal models than those found in humans.

Early success with the use of multivalency to develop a potentially more effective inhibitor of clinical symptoms induced by the influenza virus supports the suggestion that a similar approach might be effective for inhibiting infection by other microbes that are dependent on sialidase activity for part of the infectious process. However, the effectiveness of a multivalent ligand is dependent upon the sialidase having multiple ligand binding sites and knowledge of their spacing, which underscores the need to continue to gather information about their structure as well as their function(s) in the pathogen’s life cycle.

In terms of development, it is apparent that each hNEU has a role. This is especially true when looking at the central nervous system. In this case, development can be considered to be a lifelong procedure, as changes in sialidase activity and sialoglycoconjugates are continually occurring. The fact that changes that appear minor may be ongoing long before associated clinical changes become apparent will require the ongoing development of tools with which to study these changes and the ability to connect changes induced by hNEU activity with those that may occur in sialoglycoconjugate synthesis, signal transduction efficacy, and more general metabolic changes, such as those seen in lipid metabolism. Ready access to all of this knowledge will optimize the use of personalized approaches in the treatment of individuals with sialidase-related diseases.

## Figures and Tables

**Table 1:** General information about human sialidases.

Human Sialidase	Catalytic Substrate Specificity	Subcellular Distribution	pH Optimum	Examples of Associated Diseases
NEU1	β2 integrin and ICAM-1 [15]; α2,6-linked Sia [16]; oligosaccharides and short glycopeptides [17]	Intra-lysosomal [1,18]; plasma membrane [19]	4.5–4.7 [20]	Sialidosis [21,22], fibrosis [23], and cardiovascular [24]
NEU2	Oligosaccharides, glycopeptides, and gangliosides [25]	Cytosol [18]	5.6 [25]	Lipid metabolism/-motor function [26]
NEU3	Gangliosides except GM1 and GM2 [7], EGFR [27], and α2,6-linked Sia [16]	Outer surface of the plasma membrane and endosomes [7,18,28]	4.5–4.8 and 6.0–6.5 [3]	Fibrosis [9], cancer (e.g., [29,30]), neuronal function [12,14]
NEU4	Oligosaccharides, glycoproteins, and gangliosides [31]	Lysosomes, NEU4l ^1^ mitochondria and NEU4s endoplasmic reticulum [18,31,32]	3.5 is optimum but remains active over a range of pH [31]	Neuronal function [12], clearance of sialidosis and galactosialidosis products from cells ([31], recent review [33]), and inhibition of colon cancer cell motility and growth [34]

^1^ See review by Bourguet et al. [35] for an explanation of the long and short forms of hNEU4. For details on each, see the references. It can be seen that there is some overlap in substrate specificity which may be necessary due to the subcellular distribution of the sialidases and their roles in a variety of biological processes, as recently reviewed by Zhu et al. [36]. For a recent review on cellular translocation of sialidases see Aljohani et al. [37].

**Table 2:** The carbohydrate sequences of the discussed human gangliosides and potential sites of action for NEU3.

Ganglioside	Saccharide Composition
GD1α	Neu5Acα2-3^a^Galβ1-3(Neu5Acα2-6^b^)GalNAcβ1-4Galβ1-4Glcβ1-^c^
GM3	Neu5Acα2-3Galβ1-4Glcβ1-
GM2	GalNAcβ1-4(Neu5Acα2-3)Galβ1-4Glcβ1-
GM1a	Galβ1-3GalNAcβ1-4(Neu5Acα2-3) Galβ1-4Glcβ1-
GD1a	Neu5Acα2-3Galβ1-3GalNAcβ1-4(Neu5Acα2-3) Galβ1-4Glcβ1-
GT1a	Neu5Acα2-8Neu5Acα2-3Galβ1-3GalNAcβ1-4(Neu5Acα2-3)Galβ1-4Glcβ1-
GD3	Neu5Acα2-8Neu5Acα2-3Galβ1-4Glcβ1-
GD2	GalNAcβ1-4 (Neu5Acα2-8Neu5Acα2-3)Galβ1-4Glcβ1-
GD1b	Galβ1-3GalNAcβ1-4 (Neu5Acα2-8Neu5Acα2-3)Galβ1-4Glcβ1-
GT1b	Neu5Acα2-3Galβ1-3GalNAcβ1-4 (Neu5Acα2-8Neu5Acα2-3)Galβ1-4Glcβ1-
GQ1b	Neu5Acα2-8Neu5Acα2-3Galβ1-3GalNAcβ1-4 (Neu5Acα2-8Neu5Acα2-3)Galβ1-4Glcβ1-
GT3	Neu5Acα2-8Neu5Acα2-8Neu5Acα2-3Galβ1-4Glcβ1-
GT2	GalNAcβ1-4(Neu5Acα2-8Neu5Acα2-8Neu5Acα2-3)Galβ1-4Glcβ1-
GT1c	Galβ1-3GalNAcβ1-4(Neu5Acα2-8Neu5Acα2-8Neu5Acα2-3)Galβ1-4Glcβ1-

^a^ Red indicates NEU3-susceptible sialic acid residues. ^b^ Blue indicates NEU3 sialic acid moieties that cannot be cleaved until loss of terminal sugars. ^c^ Indicates linkage of sugar structure to ceramide.

**Table 5:** Examples of the effects of hNEUs in different cancers.

hNEU	Cancer	NEU Expression and Cellular Effect	Mechanism NEU Affects
1	Bladder	Increased NEU1 expression correlates with improved prognosis; increased apoptosis and reduced mobility of cancer cells [157].	Disrupted FN-integrin α5β1 interaction; inactivated Akt signaling pathway [157].
	Hepatocellular carcinoma	Tend to see increased NEU1 correlate with increased grade or stage and higher mRNA for NEU1 correlated with poorer survival, indicating that it could be used as a prognostic marker [158].	Higher NEU1 expression correlated with the inhibition of immune function, as indicated by fewer B, CD8^+^ T, NK, and T helper cells [158].
	Melanoma	Increased NEU1 enhanced proliferation and tumor progression of melanoma cells. Potential biomarker for diagnosis [159].	Enhanced expression of *CDK2* and *CD44*; decreased expression of *CASP3* and *CASP8* [159].
	Ovarian	NEU1 siRNA inhibited proliferation, enhanced apoptosis and decreased invasion by cancer cells [160].	Reduced expression of ATP5B and ATP5J [160].
2	Myeloid leukemia	Suppression: NEU2 is not normally expressed by K562 chronic myeloid leukemia cells [161].	Expression of NEU2 impairs Bcr-Abl activity, modifying signaling and making the cells more sensitive to apoptotic stimuli [161].
	Ovarian	Decreased expression, enhanced apoptosis [162].	Decreased desialylation of Atg5 decreases the autophagosome formation needed to induce anchorage-dependent cell death of ovarian cancer cells [162].
	Pancreatic ductal adenocarcinoma	Reduced expression reduced apoptosis and enhanced cell migration [163].	Induction of the overexpression of NEU2 resulted in its association with plasma membrane-associated Fas. Catalyzed release of α2-6 sialyl residues induce apoptosis. Additionally, reduced migration and invasion [163].
	Prostate	Relatively high expression of NEU2 needed for cell survival and motility [164].	Cell survival and motility required NEU2, with elevated NEU2 expression dependent upon the expression of *Runx2* and *Sp3* [164].
3	Bladder	Highly expressed, NEU3 contributes to bladder cancer cell invasiveness [30].	NEU3 activates ERK and PI3K signaling [30].
	Colon	Elevated in restricted cases of human colon cancer [29].	NEU3 inhibited apoptosis, and was accompanied by increased Bcl-2 and lactosyl ceramide, plus a decrease in caspase [29]. Can activate EGFR by catalyzing removal of its sialyl residues [27].
	Glioblastoma multiforme	Down-regulation of NEU3 expression disrupted focal adhesions, enhancing invasion and migration [155].	Reduced NEU3 resulted in reduced calpain-dependent proteolysis and GM3 [155].
	Head and neck squamous cell carcinoma	Up-regulated expression of NEU3 promoted cell mobility and invasion [154].	Enhanced phosphorylation of epidermal growth factor receptor (EGFR), and an EGFR inhibitor, AG1478 [154].
	Renal cell carcinoma	Increased NEU3 mRNA, suppresses apoptosis and promotes cell motility [165].	Increase in Gal-Cer and IL6-mediated signaling [165], controls the activation of EGFR, ß1-integrin and focal adhesion kinase (FAK)/protein kinase B (AKT) signaling [153]
4	Colon	Decreased expression is associated with increased cell invasion and proliferation [34].	Can catalyze desialylation of sialyl Lewis antigens expressed on O-glycans [34].
	Glioblastoma, most lethal brain tumor	Upregulated in glioblastoma stem cells and their maintenance [166].	Maintenance of the SHH and Wnt/β-catenin pathways that promote an embryonic stem cell-like gene expression signature [166].
	Neuroblastoma	NH4l is overexpressed in neuroblastoma cells. There is an enhanced proliferation rate and a more undifferentiated phenotype [167].	Hyperactivation of the Wnt/β-catenin signaling pathway [167].
	Ovarian carcinoma	Increased NEU4 in disseminated cells correlates with poorer survival [168].	Enhanced cancer cell motility and epithelial-mesenchymal transition desialylated EGFR, activating it [168].

## Data Availability

Data presented is available in the references cited.
